# Redox‐Mediated Artificial Non‐Enzymatic Antioxidant MXene Nanoplatforms for Acute Kidney Injury Alleviation

**DOI:** 10.1002/advs.202101498

**Published:** 2021-07-17

**Authors:** Xing Zhao, Li‐Ya Wang, Jia‐Meng Li, Li‐Mei Peng, Chun‐Yan Tang, Xiang‐Jun Zha, Kai Ke, Ming‐Bo Yang, Bai‐Hai Su, Wei Yang

**Affiliations:** ^1^ College of Polymer Science and Engineering Sichuan University State Key Laboratory of Polymer Materials Engineering Chengdu Sichuan 610065 China; ^2^ Department of Nephrology Med‐X Center for Manufacturing West China Hospital Sichuan University Chengdu 610041 China; ^3^ The First People's Hospital of Shuangliu District Chengdu 610200 China

**Keywords:** acute kidney injury, MXene, non‐enzymatic antioxidant, oxidative stress, redox

## Abstract

Acute kidney injury (AKI), as a common oxidative stress‐related renal disease, causes high mortality in clinics annually, and many other clinical diseases, including the pandemic COVID‐19, have a high potential to cause AKI, yet only rehydration, renal dialysis, and other supportive therapies are available for AKI in the clinics. Nanotechnology‐mediated antioxidant therapy represents a promising therapeutic strategy for AKI treatment. However, current enzyme‐mimicking nanoantioxidants show poor biocompatibility and biodegradability, as well as non‐specific ROS level regulation, further potentially causing deleterious adverse effects. Herein, the authors report a novel non‐enzymatic antioxidant strategy based on ultrathin Ti_3_C_2_‐PVP nanosheets (TPNS) with excellent biocompatibility and great chemical reactivity toward multiple ROS for AKI treatment. These TPNS nanosheets exhibit enzyme/ROS‐triggered biodegradability and broad‐spectrum ROS scavenging ability through the readily occurring redox reaction between Ti_3_C_2_ and various ROS, as verified by theoretical calculations. Furthermore, both in vivo and in vitro experiments demonstrate that TPNS can serve as efficient antioxidant platforms to scavenge the overexpressed ROS and subsequently suppress oxidative stress‐induced inflammatory response through inhibition of NF‐*κ*B signal pathway for AKI treatment. This study highlights a new type of therapeutic agent, that is, the redox‐mediated non‐enzymatic antioxidant MXene nanoplatforms in treatment of AKI and other ROS‐associated diseases.

## Graphical abstract

A novel non‐enzymatic antioxidant MXene nanoplatform with excellent biocompatibility and great chemical reactivity toward multiple ROS is developed to potentially scavenge the overexpressed ROS and subsequently suppress oxidative stress‐induced inflammatory response through the inhibition of NF‐*κ*B signaling pathway in the prevention and treatment of AKI and other ROS‐related diseases.

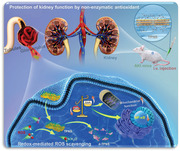

## 1 Introduction

Acute Kidney Injury (AKI),^[^ ^1^ ^]^ is one of the most common clinical diseases in hospitalized patients, especially for those in intensive care units, which has become a growing concern in healthcare worldwide.^[^ ^2^, ^3^ ^]^ AKI is commonly defined as a sudden episode of kidney failure or kidney damage that develops within a few hours or a few days,^[^ ^4^ ^]^ which is characterized by increased levels of urea nitrogen and creatinine, accompanied by a decrease in urine output.^[^ ^5^ ^]^ In general, AKI patients have higher risk of developing other health problems, such as chronic kidney disease, high potassium levels, hyperkalemia, metabolic acidosis, body fluid balance changes, or having AKI again in the future, so AKI patients suffer from high morbidity and mortality.^[^ ^6^ ^]^ Worse still, as one of the current global health threats, COVID‐19 (coronavirus disease 2019) may lead to severe kidney diseases including AKI. People who experienced COVID‐19 were reported to be at significant risk of AKI, and the likelihood of developing AKI is twice that of non‐COVID patients who have developed AKI.^[^ ^7^, ^8^ ^]^ Unfortunately, currently it has only mild treatments such as rehydration, renal dialysis, and some other supportive therapies,^[^ ^9^ ^]^ while specific therapies for AKI are urgently demanded, especially for those with COVID‐19.

During the progression of AKI, toxic reactive oxygen species (ROS) generated in excess react with a few biomolecules to cause irreversible and permanent biomolecular damage, such as DNA damage, lipid peroxidation, or protein denaturation,^[^ ^10^ ^]^ thereby triggering oxidative stress and excessive inflammatory response, and even chronic inflammatory and abrupt kidney injury.^[^ ^11^, ^12^ ^]^ Consequently, ROS is regarded as one of the critical targets in AKI treatment, and effective ROS clearance in the kidney might regulate the renal microenvironment and reduce the pathological progression of oxidative stress‐mediated AKI.^[^ ^13^ ^]^ Some molecular antioxidants such as *N*‐acetyl cysteine (NAC) have shown positive effect on AKI prevention, however, rapid excretion, poor bioavailability, and low ROS scavenging efficacy of these antioxidants limit their clinical applications.^[^ ^14^ ^]^ Therefore, it is still of a big challenge to design innovative antioxidant and anti‐inflammatory strategies to broadly and efficiently target various toxic ROS for effective treatment of AKI and other ROS‐related diseases.

For maintaining the intracellular redox homeostasis in human body, endogenous antioxidant defense system, mainly composed of enzymes (e.g., superoxide dismutase (SOD), catalase (CAT), and glutathione peroxidase (GP*_x_*), etc.) and non‐enzymatic antioxidants (e.g., glutathione (GSH), etc.), can eliminate excess ROS to prevent or reduce progressive oxidative damage.^[^ ^15^ ^]^ Although these endogenous antioxidants work out well under normal physiological conditions, they are normally insufficient to offset excessive ROS generation under pathological conditions.^[^ ^16^ ^]^ Inspired by the natural antioxidant defense system, numerous exogenous antioxidative nanomaterials have recently attracted great attention as a new type of antioxidant due to their improved stability, broad‐spectrum antioxidant capabilities, and higher ROS scavenging efficiency.^[^ ^17^, ^18^ ^]^ Typically, a plethora of synthetic enzyme‐mimicking nanomaterials (nanozymes),^[^ ^19^, ^20^, ^21^ ^]^ such as metal nanoparticles,^[^ ^22^ ^]^ metal oxide nanoparticles,^[^ ^18^, ^23^, ^24^, ^25^ ^]^ carbon‐based nanomaterials,^[^ ^26^ ^]^ biomolecules,^[^ ^27^, ^28^ ^]^ and quantum dots,^[^ ^29^, ^30^ ^]^ have demonstrated multiple ROS scavenging capability and admirable antioxidant activity.^[^ ^31^ ^]^ For example, to obtain enzyme‐mimicking nanoparticles with good biocompatibility and quick renal clearance, Deng et al.^[^ ^22^ ^]^ prepared ultrasmall Cu_5.4_O nanoparticles with multiple enzyme‐mimicking and broad‐spectrum ROS scavenging ability, which exhibited cytoprotective effects against ROS‐mediated damage and significantly improved treatment outcomes in AKI and ROS‐related diseases. Nevertheless, these artificial enzymes are always capable of catalyzing ROS generation at the same time,^[^ ^32^ ^]^ leading to potentially deleterious adverse effects, such as the induction of oxidative stress,^[^ ^33^ ^]^ activation of immune cells, and genotoxicity, which limit their clinical translation.^[^ ^34^, ^35^ ^]^ In contrast, non‐enzymatic antioxidant nanomaterials with broad‐spectrum free radical scavenging capacity enable preventing or reducing oxidative damage without apparent side effects, meanwhile they have better biocompatibility and higher potential for clinical translation.

Currently, 2D nanomaterials, as a new subtype of nanomaterials with ultrathin layer‐structured topology,^[^ ^36^ ^]^ have attracted increasing attention for biomedical applications owing to their high specific surface area and prominent physiochemical natures.^[^ ^37^, ^38^, ^39^, ^40^ ^]^ In particular, the latest nanomaterials, that are, 2D MXenes, has shown great potentials in the biomedical field serving as drugs, protein carriers, and promising theranostic nanomedicine,^[^ ^41^, ^42^, ^43^ ^]^ which is ascribed to their fascinating physiochemical properties and biological effects, such as excellent photothermal conversion,^[^ ^44^, ^45^, ^46^ ^]^ X‐ray attenuation,^[^ ^47^ ^]^ electron transparency, localized surface plasmon resonance,^[^ ^48^ ^]^ cellular endocytosis,^[^ ^49^ ^]^ and enzyme‐triggered biodegradation.^[^ ^45^ ^]^ More importantly, 2D Ti_3_C_2_ MXene holds great chemical reactivity toward ROS,^[^ ^50^ ^]^ yet it has not been systematically studied. Moreover, Ti_3_C_2_ MXene demonstrates the intrinsic characteristic of enzyme/H_2_O_2_‐responsive biodegradability, which will degrade into Ti(II) suboxide or Ti(III) suboxide or even Ti(IV) oxide (TiO_2_) with negligible risk of adverse effects after therapeutic treatment.^[^ ^49^, ^51^ ^]^ Therefore, Ti_3_C_2_ MXene would be an effective antioxidant with broad‐spectrum ROS scavenging ability and biosafety to treat a series of diseases related to ROS including AKI, however, these remain to be well explored and verified.

**Figure 1:**
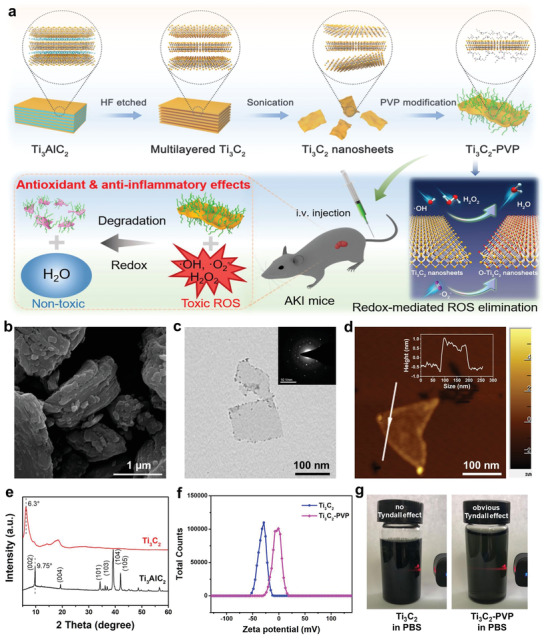
Schematic illustration of AKI treatment employing TPNS and characterization. a) Schematic of the TPNS synthesis process and their activity as redox‐mediated artificial non‐enzymatic antioxidant nanoplatforms for AKI treatment. b) SEM image of Ti_3_AlC_2_ MAX. c) TEM images of delaminated Ti_3_C_2_ nanosheets with SAED pattern (inset), and d) atomic force microscope (AFM) image of Ti_3_C_2_ nanosheets. Inset is the corresponding height profile. e) XRD patterns of Ti_3_AlC_2_ and delaminated Ti_3_C_2_ nanosheets. f) Zeta potential of Ti_3_C_2_ and Ti_3_C_2_‐PVP dispersed in water. g) Digital photographs of Ti_3_C_2_ and Ti_3_C_2_‐PVP dispersed in PBS solution.

Herein, we explore the capability of 2D Ti_3_C_2_ MXene nanosheets as artificial non‐enzymatic ROS scavengers, for highly efficient antioxidant and anti‐inflammatory effects, as well as prevention of ROS‐mediated AKI in the murine model (**Figure** **1**a). The Ti_3_C_2_ nanosheets with geometrical framework similar with previously reported flake‐like DNA frameworks which preferentially accumulate in the kidneys of AKI mice,^[^ ^28^ ^]^ are likely to enable effective renal uptake for AKI treatment. These Ti_3_C_2_ sheets exhibit improved biocompatibility and physiological stability after surface modification by polyvinylpyrrolidone (PVP) to form Ti_3_C_2_‐PVP nanosheets (TPNS), as well as enzyme/ROS‐triggered biodegradability without any obvious toxicity as evidenced both in vitro and in vivo. Moreover, they possess broad‐spectrum redox‐mediated ROS eliminating activities against various ROS including H_2_O_2_, •OH, and •O_2_^−^, allowing for alleviating the oxidative stress‐induced damage as non‐enzymatic antioxidant platforms attested by both density functional theory (DFT) calculations and in vivo and in vitro experiments. Thus, this study introduces an interesting and effective way to alleviate AKI via non‐enzymatic antioxidative protection of 2D MXene.

## 2 Results and Discussion

The 2D Ti_3_C_2_ nanosheets were synthesized via a method reported in our previous work.^[^ ^52^ ^]^ First, the Ti_3_AlC_2_ MAX phase with a layered morphology (Figure 1b) was etched with LiF/HCl solution to remove the Al layers. Then, prolonged ultrasonic delamination was employed to obtain ultrathin Ti_3_C_2_ nanosheets with reduced lateral dimensions for better uptake by cells.^[^ ^53^ ^]^ The transmission electron microscopy (TEM) image displays the ultrathin Ti_3_C_2_ nanosheets with a lateral dimension of ≈200 nm (Figure 1c), and the preserved hexagonal symmetry structure was confirmed by selected area electron diffraction (SAED) pattern. An atomic force microscopy (AFM) image of Ti_3_C_2_ nanosheets as well as AFM height profile, shown in Figure 1d, reveals that the Ti_3_C_2_ nanosheets are single‐layered or few‐layered. The X‐ray diffraction (XRD) patterns further prove the formation of delaminated Ti_3_C_2_ nanosheets as evidenced by leftward shift of the (002) peak and the disappeared characteristic peaks of Ti_3_AlC_2_ (Figure 1e).

Although the prepared Ti_3_C_2_ nanosheets are hydrophilic and water‐dispersible, they show pretty poor stability in salt solution, resulting in severe aggregation and poor biocompatibility. Therefore, the Ti_3_C_2_ nanosheets were surface‐modified with PVP chains to improve their colloidal stability under physiological conditions because of steric hindrance by the macromolecular chains, as proved by the increase of zeta potential of Ti_3_C_2_‐PVP nanosheets aqueous dispersion (Figure 1f). The typical C═O and C—N signals in the FTIR spectrum of Ti_3_C_2_‐PVP indicate the successful PVP modification onto the surface of Ti_3_C_2_ (Figure S1a, Supporting Information). The PVP content in Ti_3_C_2_‐PVP was calculated to be 17.4% according to the thermogravimetric analysis (Figure S1b, Supporting Information). As a result, the TPNS exhibited excellent dispersity in PBS solution with obvious Tyndall scattering effect, while such a phenomenon was missing in the Ti_3_C_2_‐dispersed PBS solution (Figure 1g). Furthermore, the similar hydrodynamic size distribution of Ti_3_C_2_‐PVP in various solvents, including water, PBS, FBS, and DMEM, indicated the excellent colloidal stability of Ti_3_C_2_‐PVP under physiological conditions (Figure S2, Supporting Information).

**Figure 2:**
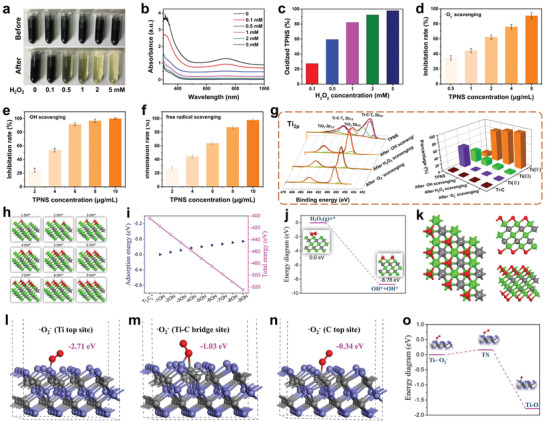
Experiments and theoretical calculations demonstrating the redox‐mediated ROS scavenging mechanism of TPNS. Digital photographs (a) and UV–vis–NIR absorption spectra (b) of TPNS after incubation with different concentrations of H_2_O_2_. c) Relatively oxidized TPNS calculated from (b). d–f) ROS inhibiting efficiency of TPNS at different concentrations for •O_2_^−^, •OH, and ABTS free radical, respectively. g) XPS patterns of TPNS in the Ti 2p region before and after various ROS scavenging (left), and statistical graph of different valence states of Ti in the TPNS before and after various ROS scavenging (right). h) The geometrically optimized structures of Ti_3_C_2_ upon continuous •OH radical attack. i) The adsorption energy and total energy of the system upon continuous •OH radical attack. j) Energy diagram for the conversion of H_2_O_2_ on the surface of Ti_3_C_2_. k) Geometrically optimized nanostructure of oxidized Ti_3_C_2_ observed from different angles. l–n) Three different •O_2_^−^ adsorption sites onto Ti_3_C_2_ with optimized adsorption energy. o) Energy diagram for oxidation of Ti_3_C_2_ upon •O_2_^−^ attack.

Considering the crucial role of ROS in AKI prevention and treatment, a systematic evaluation of the potential of TPNS nanosheets as powerful ROS scavengers was performed. Three representative ROS, including H_2_O_2_ (hydrogen peroxide), •O_2_^−^ (superoxide anion radicals), and •OH (hydroxyl radicals) were used to investigate multiple ROS elimination capabilities of TPNS. The color of TPNS suspension gradually became weaker as the concentration of H_2_O_2_ increased from 0.1 up to 5 × 10^−3^ m (**Figure** **2**a), demonstrating excellent H_2_O_2_ scavenging ability of TPNS. The decreased UV–Vis–NIR absorption was due to the oxidation of Ti_3_C_2_ by H_2_O_2_ (Figure 2b),^[^ ^51^ ^]^ and only 0.1 × 10^−3^ m H_2_O_2_ can easily cause Ti_3_C_2_ oxidation (Figure 2c), which indicates that the TPNS has great chemical reactivity toward H_2_O_2_. The antioxidative activity of TPNS toward radicals of •O_2_^−^ and •OH show that more than 90% •O_2_^−^ and almost all of •OH were scavenged with the addition of low concentration TPNS (Figure 2d,e and Figure S3, Supporting Information), indicating excellent scavenging capacity of TPNS against •O_2_^−^ and •OH. Besides, the excellent antioxidative properties of TPNS were confirmed by an ABTS radical scavenging assay (Figure 2f).^[^ ^54^ ^]^

Owing to the intrinsic reducibility of Ti_3_C_2_ MXene,^[^ ^51^, ^55^ ^]^ Ti_3_C_2_ nanosheets are oxidized into Ti‐based oxides when ROS is eliminated confirmed by XPS patterns of Ti_2p_ (Figure 2g), which contributes to the great ROS scavenging performance of TPNS. Specifically, two peaks of Ti‐C‐T*_x_* 2p_1/2_ and Ti‐C‐T*_x_* 2p_3/2_ almost disappeared after ROS scavenging and the intensity of TiO_2_ peaks increased significantly, indicating intensive oxidation of Ti_3_C_2_ during ROS scavenging. Besides, the statistical graph of different valence states of Ti in TPNS before and after various ROS scavenging shows that the oxidized Ti (IV) species increase significantly, while the Ti‐C, Ti (II), and Ti (III) species decrease obviously, thus TPNS can be entirely oxidized by various ROS.

Based on the above results, we propose the redox‐mediated ROS scavenging mechanism, that is, ROS were scavenged by Ti_3_C_2_ through the redox reaction between ROS and Ti_3_C_2_, which is verified by DFT calculations. The initial total energy of the geometrically optimized single‐layered Ti_3_C_2_ (*) is −404.012 eV (Figure S4, Supporting Information). Comparing with the [CTi_3_] and Ti atom site, the [Ti_3_C] site is calculated as most energy‐favorable site for •OH adsorption with an adsorption energy of −6.00 eV (Figure S5, Supporting Information). From the investigated 3 × 3 lattice, nine [Ti_3_C] sites can be utilized to adsorb •OH. Upon the attack of •OH radicals, [Ti_3_C] sites are occupied one by one (Figure 2h), and total system energy continues to decrease until all [Ti_3_C] sites are completely occupied (Figure 2i). The H_2_O_2_ molecules are also easily adsorbed on the surface of Ti_3_C_2_ and converts into two hydroxyl groups attached to Ti_3_C_2_ nanosheet as indicated in Figure 2j. Gradually, the hydroxyl groups could be dehydrated to form an additive O layer on top Ti layer as shown in Figure 2k. Further simulation of the adsorption process of •O_2_^−^ on the surface of Ti_3_C_2_ nanosheets reveals that the Ti top sites are the most favorable sites for •O_2_^−^ adsorption with an adsorption energy of −2.72 eV comparing with the Ti—C bridge site (−1.03 eV) and C top site (−0.34 eV) (Figure 2l–n). Besides, the Ti_3_C_2_ can easily adsorb •O_2_^−^ to generate oxygenic nanosheets (TiO*_x_* species), as shown in Figure 2o. Therefore, the DFT calculation results are consistent with the XPS results, indicating the surface oxidation of Ti_3_C_2_ nanosheets contributing to their great ROS scavenging ability. Therefore, theoretical calculations and experimental results indicate that the ROS scavenging mechanism of TPNS is mainly caused by the inherent reducibility of Ti_3_C_2_ MXene, which can easily induce redox reactions between Ti_3_C_2_ and various ROS.

**Figure 3:**
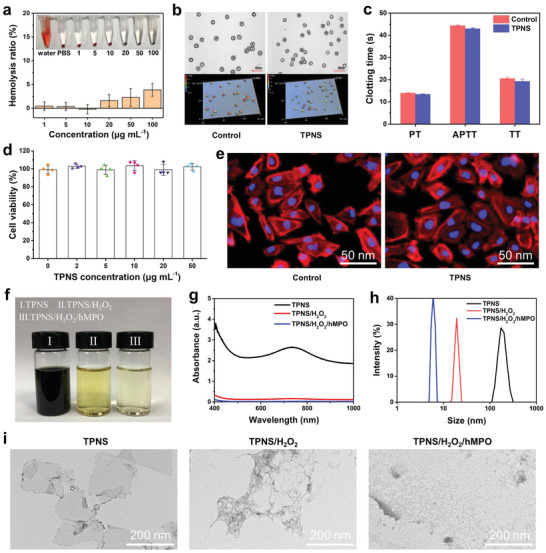
In vitro biocompatibility and biodegradability assessment of TPNS. a) In vitro hemolysis test of TPNS. Insets are digital photographs of hemolysis for TPNS with different concentrations. b) The 3D microscope images of RBCs before and after incubation with TPNS. c) Clotting time of blood treated with TPNS. d) Cell viability for HK‐2 after incubation with TPNS for 24 h. e) Cytoskeleton staining of HK‐2 cells before and after incubation with TPNS at 50 µg mL^−1^ for 24 h. f–i) Digital photographs, UV–Vis–NIR absorption spectra, dynamic light scattering analysis, and TEM images of TPNS after various treatments, respectively. In (a), (c), and (d), data represent mean ± SD from three (a,c) or four (d) independent replicates.

Red blood cells (RBCs) compatibility is essential to ensure the safety of intravenous administration. As shown in **Figure** **3**a, the hemolysis rates of TPNS nanosheets are less than 5% even at a high dose of 100 µg mL^−1^, which is in line with the ASTM standard (ASTM F756‐2008).^[^ ^56^ ^]^ In addition, morphological analysis shows that the RBCs show no change in morphologies after incubation with TPNS compared with the control group (Figure 3b and Figure S6, Supporting Information), demonstrating the excellent RBCs compatibility of TPNS. Furthermore, the clotting time of blood incubated with TPNS show little difference with the control group, which indicates the negligible effect of TPNS on coagulation time. Next, the CCK‐8 (cell counting kit‐8) assay results show no significant difference in cell viability as the concentration of TPNS increases. Meanwhile, human renal tubular epithelial (HK‐2) cells after incubating with 50 µg mL^−1^ TPNS for 24 h showed the same spindle cytoskeleton morphology as the control group (Figure 3e), revealing no cytotoxicity of TPNS to HK‐2 cells. These results indicate the good biocompatibility of TPNS.

Previously widely studied inorganic nanozymes usually have poor biodegradability and long‐term retention in the body. However, Wang et al.^[^ ^40^ ^]^ reported the use of black phosphorus nanosheets (BPNSs) as ROS scavengers to cure AKI in mice. Importantly, black phosphorus nanosheets (BPNSs) exhibited excellent biodegradability, and can be oxidized to non‐biotoxic P*_x_*O*_y_* ions. Similarly, the as‐prepared TPNS exhibits the intrinsic biodegradability triggered by enzyme/H_2_O_2_, which can ensure excretion from the body after fulfilling their therapeutic administration.^[^ ^45^, ^57^ ^]^ Human myeloperoxidase (hMPO), a powerful enzyme which catalyzes the production of hypochlorous acid and contributes to the degradation of various target substances in the human body,^[^ ^58^, ^59^ ^]^ was employed to evaluate the biodegradability of TPNS. As shown in Figure 3f, the TPNS nanosheets treated with H_2_O_2_/hMPO will degrade over time and the suspension becomes transparent, which is in accordance with the disappearance of light absorbance of the TPNS in the UV–Vis–NIR absorbance spectra (Figure 3g) after H_2_O_2_/hMPO treatment. Furthermore, dynamic light scattering (DLS) measurements show a drastic reduction in the size of H_2_O_2_/hMPO‐treated TPNS compared to the control (Figure 3h). The morphology of TPNS nanosheets in Figure 3i further confirms the degradation of the original planar structure of nanosheets after treatment with H_2_O_2_/hMPO. These nanodots from the degradation of the TPNS nanosheets can be rapidly excreted from the body, thus indicative of negligible long‐term toxicity of TPNS nanosheets. Furthermore, the intracellular degradation tests confirm the TPNS can be degraded by the cells (Figure S7a, Supporting Information), and the excretion pathways including urine and feces show that nearly 35% of Ti content was excreted out of mice by 24 h post intravenous injection (Figure S7b, Supporting Information), which exhibits the possibility of TPNS being excreted out of the body via the urine and feces. Therefore, TPNS overall shows good biocompatibility and biodegradability as potential clinically demanding biomaterials.

**Figure 4:**
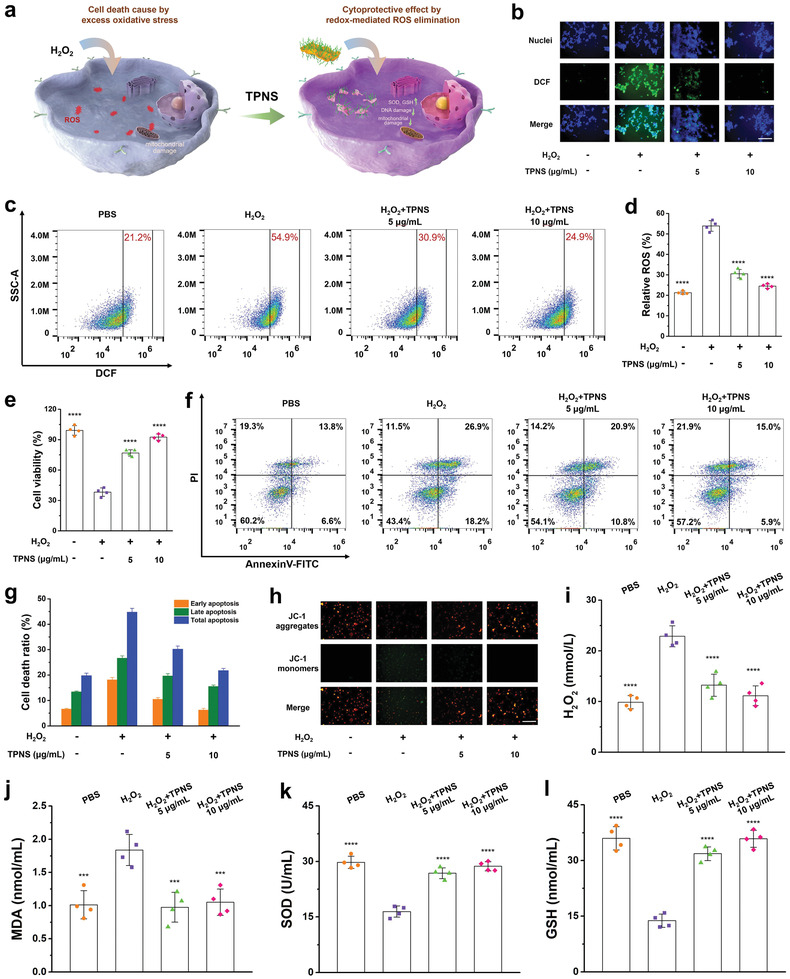
ROS scavenging activity of TPNS in vitro. a) Schematic illustration of the cytoprotective effect of TPNS by redox‐mediated ROS elimination. b) Representative ROS staining of HK‐2 cells after different treatments. Scale bar: 200 µm. c) Flow‐cytometry‐based assay of ROS levels of HK‐2 cells after different treatments. d) Statistical analysis for ROS levels of HK‐2 cells after different treatments. e) Cell viabilities of HK‐2 cells after different treatments. f) Flow‐cytometry‐based apoptosis assay of cell apoptosis distribution in HK‐2 cells after different treatments. g) Statistical analysis of death ratios in HK‐2 cells after different treatments. h) Changes of the mitochondrial membrane potential in HK‐2 cells after different treatments. Scale bar: 200 µm. i) H_2_O_2_, j) MDA, k) SOD, and l) GSH levels measured in HK‐2 cells under different treatment conditions. Statistical significance compared with the H_2_O_2_ group is shown (****p < 0.0001; ***p < 0.001). In (d–e), (g), and (i–l), data represent mean ± SD from four independent replicates.

Given that the TPNS shows great antioxidative properties and excellent biocompatibility, it is highly potent to exhibit the cytoprotective effect through redox‐mediated ROS elimination in vitro (**Figure** **4**a). Since renal tubules are easily damaged by excess oxidative stress,^[^ ^60^ ^]^ protecting renal tubules from ROS damage in the early stage of AKI would be necessary to reduce renal dysfunction. Following this consideration, we first used human renal tubular epithelial cells (HK‐2) cells to evaluate the cytoprotective effects of TPNS nanosheets against ROS damage. Upon H_2_O_2_ treatment, the intracellular ROS level increased significantly, while the cells pretreated with different concentrations of TPNS exhibited significantly decreased intracellular ROS levels (Figure 4b). Further quantitative analysis of the ROS scavenging effects of TPNS for HK‐2 via flow cytometry (Figure S8, Supporting Information) also confirmed the decreased intracellular ROS level with the introduction of TPNS (Figure 4c,d). High ROS levels in HK‐2 cells cause oxidative stress, thereby disrupting mitochondrial function and inducing cell death, whereas low‐concentration TPNS potentially decreased the intracellular ROS content and protected the cells against ROS damage (Figure 4e).

As it is reported that cell apoptosis is closely associated with AKI,^[^ ^61^ ^]^ the cytoprotective effect of TPNS against cell apoptosis induced by oxidative stress was further examined via flow cytometry apoptosis assay (Figure S9, Supporting Information). As illustrated in Figure 4f, the H_2_O_2_ group demonstrated the highest level of apoptosis, emphasizing the significant effect of excessive ROS on the pathogenesis of apoptosis. After the addition of TPNS, cell apoptosis was obviously inhibited depending on the TPNS dosage. Statistical analysis in Figure 4g quantitatively showed that the addition of TPNS significantly reduced the ratios of early apoptosis and late apoptosis for HK‐2 cells, further confirming the cytoprotective properties of TPNS, which is ostensibly caused by reducing intracellular ROS level with TPNS nanosheets. Mitochondria membrane potential (MMP) is a key parameter of mitochondria function and the decrease of MMP was generally considered as a mark of early apoptosis.^[^ ^62^, ^63^ ^]^ JC‐1 staining was introduced to visually examine the change in the MMP, which is determined by the transition between JC‐1 aggregation (red fluorescence) and JC‐1 monomer (green fluorescence).^[^ ^64^ ^]^ The intensity of the green fluorescence in cells treated with H_2_O_2_ was significantly increased, while the intensity of the green fluorescence was great suppressed with the addition of TPNS (Figure 4h), indicating the contribution of TPNS to the mitochondria function recovery through the inhibition of toxic ROS.

We further conducted a series of biochemical detection to investigate the effects of TPNS on intracellular redox homeostasis at the cellular level. The intracellular H_2_O_2_ was first examined to evaluate the level of intracellular ROS. As illustrated in Figure 4i, the treatment of cells with TPNS caused significantly decreased level of intracellular H_2_O_2_, indicating the excellent intracellular ROS scavenging properties of TPNS. The intracellular MDA, which reflects the level of lipid peroxidation, was evaluated to further demonstrate that TPNS could scavenge ROS and consequently protect cells from lipid peroxidation damage. Then, we evaluated the status of intracellular antioxidant system by measuring the level of SOD and GSH. As shown in Figure 4k,l, the levels of SOD and GSH in cells after TPNS treatment were similar to those of the control group, while the H_2_O_2_ group showed reduced levels of SOD and GSH, demonstrating that TPNS nanosheets acted as effective ROS scavengers to restore the intracellular antioxidant capability.

**Figure 5:**
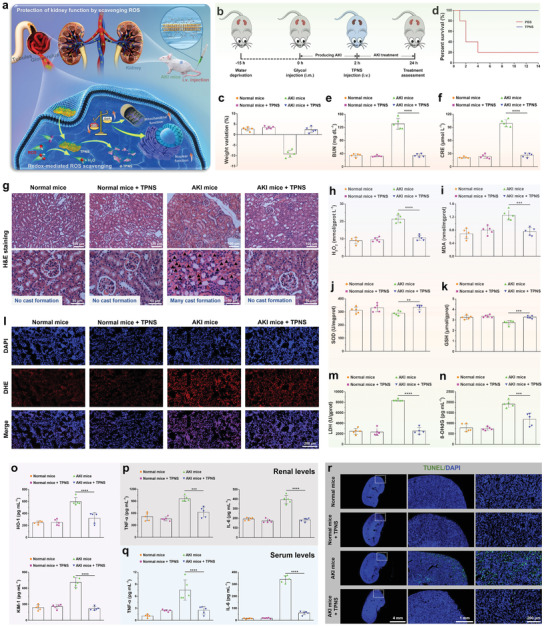
Therapeutic efficiency of TPNS for AKI mice in vivo. a) Schematic illustration of TPNS in protection of kidney function via scavenging ROS. b) Schematic illustration for establishing AKI model and their treatment with TPNS. c) Changes in weight and d) percent survival of AKI mice under different treatment conditions. e) BUN and f) CRE levels in serum from AKI mice after different treatments. g) Images of H&E‐stained renal tissues in each group. Triangles indicate the cast formation. h) H_2_O_2_, i) MDA, j) SOD, and k) GSH levels of renal tissues in each group. l) DAPI (blue fluorescence) and dihydroethidium (red fluorescence) staining of renal tissues in each group. m–o) Expression levels of biomarkers, including LDH, 8‐OHdG, HO‐1, and KIM‐1 of renal tissues in each group. p) Renal levels and q) serum levels of TNF‐*α* and IL‐1*β*. r) TUNEL fluorescence images of TUNEL‐stained kidney sections from each group. Statistical significance compared with the AKI group is shown (****p < 0.0001; ***p < 0.001; **p < 0.01). In (c), (e,f), and (h–q), data represent mean ± SD from five independent replicates.

Before in vivo experiments, the biodistribution of TPNS nanosheets in main organs at different time points was investigated after intravenous injection of cy5.5‐loaded TPNS on AKI‐bearing mice. Cy5.5‐loaded TPNS was constructed by the hydrogen bond interaction between cy5.5 molecules and the surface termination groups (such as —OH, O, and F) of Ti_3_C_2_ nanosheets. The stability test of cy5.5‐TPNS fluorescence signal showed a 76.3% fluorescence retention of cy5.5‐TPNS after 12 h incubation in solution (Figure S10, Supporting Information). Then, fluorescence imaging of main organs showed that the fluorescence signal of cy5.5 in kidney tissues was the highest at 5 min post‐injection, demonstrating that TPNS had preferential accumulations in the kidneys. The cy5.5 was gradually released from cy5.5‐TPNS, and the fluorescence signal showed a gradually decreasing trend from 5 min to 12 h post‐injection, while the maximum renal uptake of TPNS was found at 12 h post‐injection (Figure S11, Supporting Information). The effective renal uptake of the TPNS in AKI mice makes the highly efficient AKI therapy possible. Encouraged by the excellent in vitro ROS scavenging capability and in vivo biodistribution of TPNS, we concluded that the potential accumulation of TPNS in kidneys could be capable of the effective protection of renal function from ROS damage and curing AKI (**Figure** **5**a). As illustrated in Figure 5b, the AKI model was first established in mice through the intramuscular injection of 50% glycerol into dehydrated healthy mice, which triggered accumulated rhabdomyolysis‐related damage, causing oxidative stress‐related renal dysfunction.^[^ ^65^ ^]^ Subsequently, intravenous injection of TPNS nanosheets was performed to validate the potential feasibility of TPNS as an artificial non‐enzymatic antioxidant for treating ROS‐related diseases. AKI mice treated with TPNS showed the similar weight variation as the healthy mice, while AKI mice treated with PBS underwent severe weight loss at 24 h post‐injection (Figure 5c). Moreover, 80% of PBS‐treated AKI mice died within 4 days, whereas all AKI mice administered with TPNS survived for more than 14 days (Figure 5d). Two clinical renal function indicators including serum creatinine (CRE) and blood urea nitrogen (BUN) levels of TPNS‐treated AKI mice were lower than those in the AKI mice treated with PBS (Figure 5e,f),^[^ ^66^ ^]^ confirming the excellent therapeutic effect of TPNS for restoring the kidney excretory function. Excessively denatured proteins deposited in the tubules would potentially form cast structures, which are generally regarded as effective diagnostic indicators of kidney diseases. Many casts (indicated as triangles) and damaged renal tubules can be observed in the kidney section of AKI mice, while no obvious damage was found for the TPNS‐treated group (Figure 5g), suggesting that TPNS can effectively suppress the damage of renal tissues. Long‐term assessment showed the CRE and BUN levels of AKI mice after treatment with TPNS for 14 days are similar as those of normal mice, and no damage was found from H&E‐stained renal tissue of AKI mice within 14 days after treatment of TPNS (Figure S12, Supporting Information).

Furthermore, to understand the mechanism of TPNS against AKI, the ROS levels and antioxidant activities in renal tissues were measured by biochemical detection. Compared to AKI mice treated with PBS, the TPNS‐treated AKI mice showed significantly reduced H_2_O_2_ and MDA levels (Figure 5h,i). Moreover, the renal SOD and GSH activities in the TPNS‐treated AKI mice were similar to those in normal mice, while a slight decrease in SOD and GSH activities were found in the AKI treated with PBS (Figure 5j,k). Serum levels of ROS and SOD activities further demonstrated the same tendency as those in renal tissues (Figure S13, Supporting Information). These results verify that TPNS could act as antioxidants to maintain the antioxidant activities for protecting renal tissues.

Dihydroethidium (DHE) staining of kidney sections for superoxide generation was done to further perform functional investigation of TPNS against AKI. ROS production of renal tissues was significantly inhibited after TPNS treatment (Figure 5l). Furthermore, the LDH level (lactate dehydrogenase, an important biomarker of cell damage in the kidney) in AKI mice treated with TPNS was similar to that in healthy mice (Figure 5m), while a significantly elevated level of LDH was found in AKI mice treated with PBS, suggesting that TPNS could protect renal cells in AKI mice. Additionally, the 8‐hydroxy‐2'‐deoxyguanosine (8‐OHdG) level in renal tissues, a biomarker of DNA damage was also measured, demonstrating that TPNS can significantly inhibit the DNA damage (Figure 5n). Besides, the AKI mice treated with TPNS showed significantly reduced levels of kidney injury biomarkers including HO‐1 and KIM‐1 (Figure 5o),^[^ ^67^, ^68^ ^]^ which are consistent with the BUN and CRE results. Furthermore, to assess the level of systemic inflammatory response caused by AKI, both the renal and serum levels of inflammatory factors including interleukin‐6 (IL‐6) and tumor necrosis factor‐*α* (TNF‐*α*) were measured by Elisa assay (enzyme‐linked immunosorbent assay). As shown in Figure 5p,q, compared with PBS‐treated AKI mice, AKI mice treated with TPNS exhibited significantly reduced levels of inflammatory factors both in the renal tissues and serum, indicating that TPNS can inhibit the immune response during AKI. Finally, TUNEL staining of renal tissues in each group revealed anti‐apoptotic property of TPNS against AKI (Figure 5r).

To assess the toxicity of TPNS nanosheets in vivo, healthy mice were intravenously injected with TPNS nanosheets at a high dose. Then the primary organs (heart, liver, spleen, lung, and kidneys) and blood samples were collected at 24 h post‐injection. Kidney sections of glomeruli, tubules, and collecting ducts showed no obvious tissue damage compared with the control group (Figure S14, Supporting Information), suggesting no renal toxicity of TPNS. Additionally, no adverse effects were found in the other organs after 24 h post‐injection of TPNS nanosheets (Figure S15, Supporting Information). Moreover, the liver function (Figure S16a, Supporting Information), kidney function (Figure S16b, Supporting Information), and hematology indices (Figure S16c, Supporting Information) of mice injected with TPNS nanosheets were also normal by comparison with the control group. All of these confirmed the excellent biocompatibility and negligible toxicity in vivo of TPNS, thus having great potential in clinical applications.

**Figure 6:**
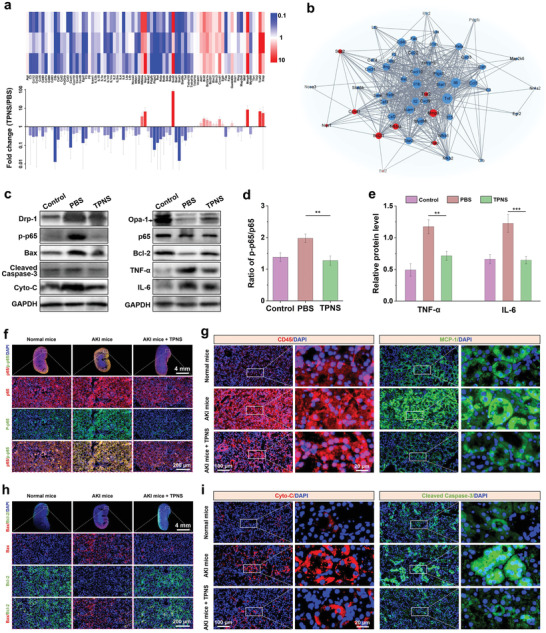
Therapeutic mechanisms of TPNS on AKI. a) PCR array heat map and fold change of differentially expressed genes in the NF‐*κ*B signaling pathway. b) PPI network (protein–protein interaction) of differentially‐expressed genes (fold change ≥ 2 or ≤ 1/2) involved in the NF‐*κ*B signaling pathway. c) Western blotting analysis of protein levels in renal tissues from different groups. Quantitative expression levels of d) p‐p65/p65 and e) inflammatory factors in renal tissues from different groups. Immunofluorescence staining of f) NF‐*κ*B and g) CD45 and MCP‐1 levels in kidney sections from different groups. Immunofluorescence staining of h) Bax and Bcl‐2, and i) Cyto‐C and Cleaved Caspase‐3 levels in kidney sections from different groups. Statistical significance compared with the AKI group is shown (***p < 0.001; **p < 0.01). In (a), and (d,e), data represent mean ± SD from three independent replicates.

To further explore the underlying therapeutic mechanisms of TPNS against AKI, we performed the transcriptomics analysis. It has been reported that excessive ROS could cause oxidative stress‐induced tissue damage,^[^ ^69^ ^]^ thereby triggering severe inflammatory responses throughout the NF‐*κ*B signaling pathway.^[^ ^70^ ^]^ Notably, several important genes related to inflammatory factors, including TNF, IL‐6, IL‐2, and IL‐1*β*, were significantly downregulated after the TPNS treatment, indicating that the therapeutic mechanism of antioxidant and anti‐inflammatory protection occurs by inhibiting NF‐*κ*B signaling pathway. The significantly‐changed genes related to NF‐*κ*B signaling pathway were used for the PPI network (protein–protein interaction) analysis. As shown in **Figure** **6**b, the neighboring proteins linked to dominant proteins include IL‐6, IL‐1*β*, etc., emphasizing the important roles of these genes in suppressing the inflammatory responses after TPNS treatment.

Further western blotting (WB) analysis of several important proteins in renal tissues was performed to comprehend the mechanism of renal protection of TPNS (Figure 6c). The significantly decreased phosphorylation of NF‐*κ*B after TPNS treatment (Figure 6d), indicates the inhibition of NF‐*κ*B signaling pathway in the TPNS‐treated AKI mice, consistent with the aforementioned transcriptomics analysis results. Besides, the downstream inflammatory factors of NF‐*κ*B pathway including IL‐6 and TNF‐*α* were significantly downregulated after TPNS treatment (Figure 6e), and the expression trend was in accordance with the aforementioned Elisa results regarding IL‐6 and TNF‐*α* protein levels (Figure 5p,q), respectively. We also studied the effect of TPNS on apoptosis‐related proteins expression. Mitochondrial fragmentation occurs before obvious renal tubular damage or cell death, and AKI is related to excessive mitochondrial fission compared with fusion.^[^ ^71^ ^]^ The Drp‐1 (dynamin‐related protein 1), which mediates mitochondrial fission, and one of the fusion‐meditated proteins (Opa‐1) was detected. The downregulated Drp‐1 and upregulated Opa‐1 in the TPNS group verify that TPNS could effectively regulate mitochondrial dynamic balance (Figure S17a, Supporting Information), which further promoted the activation of Bcl‐2 proteins (anti‐apoptotic protein) and simultaneously caused the inhibition of Bax proteins (pro‐apoptotic protein) (Figure S17b, Supporting Information). In addition, the apoptotic factor, Cytochrome C (Cyto‐C), and apoptotic executive proteins caspase3 activated by Cyto‐C, both were downregulated after TPNS treatment (Figure S17c, Supporting Information), confirming that TPNS could protect renal cells from inflammation‐induced damage and inhibit cell apoptosis.

The phosphorylation of I*κ*B (NF‐*κ*B inhibitor) could cause the dissociation and further activation of NF‐*κ*B from NF‐*κ*B‐I*κ*B complex. The activated NF‐*κ*B would transfer into nucleus to achieve the transcriptional functions. Immunofluorescence staining confirmed significantly decreased expression of p‐I*κ*B after the TPNS treatment (Figure S18, Supporting Information), further resulting in reduced nuclear accumulation of p‐NF‐*κ*B (Figure 6f), which was consistent with the aforementioned WB analysis result. Furthermore, the expression of CD45 (leukocyte common antigen), MCP‐1 (monocyte chemotactic protein 1), and F4‐80 (macrophage marker) were decreased in the TPNS‐treated AKI mice (Figure 6g and Figure S19, Supporting Information), indicating that TPNS could suppress inflammatory response in AKI mice through the inhibition of NF‐*κ*B signaling pathway. The significantly decreased expression of inflammatory factors including TNF‐*α*, IL‐6, and IL‐1*β* (Figure S20, Supporting Information), further confirmed the suppression of inflammatory response after TPNS treatment. In addition, the suppression of inflammatory factors significantly reduced the expression of Bax (Figure 6h), further causing the decreased Cyto‐C and cleaved caspase‐3 protein levels as indicated by the immunofluorescence staining analysis (Figure 6i). The overall results indicated that TPNS could reduce the oxidative stress in AKI through effective ROS scavenging, further suppressing the inflammatory response by the inhibition of NF‐*κ*B signaling pathway and protecting the renal tissues against AKI.

## 3 Conclusion

In summary, a novel artificial non‐enzymatic antioxidant MXene‐based nanoplatform for effective AKI therapy in living animals was presented in this work. Both in vitro and in vivo experiments demonstrated that the prepared TPNS nanosheets exhibited broad‐spectrum redox‐mediated ROS eliminating activities against oxidative stress‐induced damage. Additionally, the TPNS nanosheets displayed characteristic ROS‐responsive biodegradation and excellent long‐term biocompatibility. With effective renal uptake, great ROS scavenging activity, these TPNS nanosheets exhibited excellent therapeutic efficiency of ROS‐induced AKI, as demonstrated by CRE and BUN measurement of nitrogenous wastes, pathological staining, biomarkers detection, transcriptomics analysis, western blotting analysis, and immunofluorescence staining of kidney tissues. Overall, the synthesized TPNS nanosheets with excellent biocompatibility and robust ROS scavenging capability could represent a promising non‐enzymatic antioxidant to reduce oxidative stress for the prevention and treatment of AKI.

## Conflict of Interest

The authors declare no conflict of interest.

## Supporting information

Supporting Information

## Data Availability

Research data are not shared.
