# Supplementary material for: Redox‐Mediated Artificial Non‐Enzymatic Antioxidant MXene Nanoplatforms for Acute Kidney Injury Alleviation
Source: Adv Sci (Weinh). 2021 Jul 17;8(18):2101498. doi: 10.1002/advs.202101498 (PMC8456282; doi:10.1002/advs.202101498)
Supplement: Supplementary file 1 — Supporting Information [file ADVS-8-2101498-s001.pdf]

© 2021 Wiley-VCH GmbH

ADVANCED SCIENCE
Open Access

Supporting Information

for *Adv. Sci.,* DOI: 10.1002/advs.202101498

Redox-Mediated Artificial Non-Enzymatic Antioxidant MXene Nanoplatforms for Acute Kidney Injury Alleviation

*Xing Zhao, Li-Ya Wang, Jia-Meng Li, Li-Mei Peng, Chun-Yan Tang, Xiang-Jun Zha, Kai Ke, Ming-Bo Yang, Bai-Hai Su*and Wei Yang**

Supporting Information

Redox-Mediated Artificial Non-Enzymatic Antioxidant MXene Nanoplatforms for Acute Kidney Injury Alleviation

Xing Zhao[†]*, Li-Ya Wang*[†]*, Jia-Meng Li, Li-Mei Peng, Chun-Yan Tang, Xiang-Jun Zha, Kai Ke, Ming-Bo Yang, Bai-Hai Su*[*] *and Wei Yang*[*]

Experimental Section

***Synthesis of Ti_3C_2-PVP nanosheets*:** Layered ternary carbide (Ti_3AlC_2) MAX phase powder was procured from 11 Technology Co., LTD. LiF powder (2g, Alfa Aesar, 98+%) was dissolved in 20 ml 9M HCl (Aladdin Biochemical Technology Co., Ltd.) in a Teflon container under magnetic stirring for 10 minutes to ensure the dissolution of LiF. Then, 2 g Ti_3AlC_2 powder was slowly added into the LiF/HCl etching solution, and the mixture reacted for 2 d at 35 °C under magnetic stirring. The resultant $Ti_3C_2T_x$ suspension was repeatedly washed with deionized water and centrifuged to remove the residual LiF. The delamination of $Ti_3C_2T_x$ was conducted by prolonged sonication treatment under N_2 atmosphere for 1 h, and followed by centrifugation for 30 min at 3500 rpm to obtain a homogeneous supernatant with delaminated Ti_3C_2 (MXene). The concentration of MXene colloidal solution was determined by filtering a known volume of the solution on a polypropylene filter (Celgard 3501 coated PP) and measuring the weight of the resulting freestanding film after vacuum drying. For the surface modification of Ti_3C_2 nanosheets, 10 mL Ti_3C_2 (5 mg mL^{-1}) and 200 mg PVP were dissolved into 100 mL of anhydrous ethanol and refluxed at 50 °C for 4 h. The excess PVP was removed by centrifugation at 10 000 rpm for 30 min. Afterward, the resulting Ti_3C_2-PVP was washed with ethanol and water for further use.

***Characterizations*:** Scanning electron microscopy (SEM) images were recorded using a JSM-5900LV SEM microscope (JEOL, Japan) at an accelerating voltage of 15 kV. Transmission electron microscopy (TEM) images were obtained on a high-resolution transmission electron microscope

(Tecnai G2 F20S-TWIN) equipped with a field emission gun operating at 200 kV. X-ray photoelectron spectrum (XPS) was performed on an XSAM800 X-ray Photoelectron Spectrometer (Kratos Company, UK) with the Al Kα radiation (hv =1486.6 eV). X-ray diffraction (XRD) analysis was carried out on a Japan Rigaku X-ray diffractometer (UItima Ⅳ) from 2θ angle of 5 ° to 45 ° using Cu Kα radiation ($\lambda = 0.154056$ nm) at a scanning speed of 10° min^{-1}. The UV-Vis-NIR absorption was measured in the wavelength range of 300-1000 nm using an ultraviolet-visible near-infrared (UV-vis-NIR) spectrophotometer (UV3600, Shimadzu, Japan). Tapping mode atomic force microscopy (AFM) images were recorded with atomic force microscope (AFM, Multimode 8, Bruker Corporation), and samples were prepared by dropping the solution on the substrate of mica. Dynamic light scattering (DLS) and Zeta potentials were measured with a Nano-ZS Zetasizer.

***H_2O_2* scavenging assays:** H_2O_2 scavenging activity of TPNS was tested by incubating TPNS suspension with various concentrations of H_2O_2 (0.5-5 mM) at 37 °C for 1 h. TPNS was oxidized by H_2O_2 to form oxidized TPNS with decreased Vis-NIR absorption. Then, the relative percentage of oxidized TPNS was determined by the absorbance peak in 735 nm of the TPNS suspension.

***•O_2^-* scavenging assays:** The superoxide anion (•O_2^-) scavenging activity was assessed using a superoxide anion assay kit (Nanjing Jiancheng Bioengineering Institute, Nanjing, China) according to the manufacturer’s instructions. Different concentrations of TPNS (0.5-8 μg mL^{-1}) were added to the working solution. The absorbance at 550 nm was measured using a multiple plate reader after standing for 10 min.

***•OH scavenging assays*:** The hydroxyl radical (•OH) scavenging activity was assessed using a hydroxyl radical assay kit (Nanjing Jiancheng Bioengineering Institute, Nanjing, China) based on the TMB chromogenic method. Different concentrations of TPNS (2–10 μg mL^{-1}) were added to the working solution. After incubating for 1 min at 37 °C, Fenton reagent was added to each microplate well, and the absorbance at 550 nm was measured using a multiple plate reader after standing for 30 min.

The ESR spectroscopy signal was measured by a Bruker EMX plus spectrometer (Bruker, Germany). Typically, 100 μM $FeSO_4$, 1 mM H_2O_2, and different concentration of TPNS (0, 2, 5 μg mL^{-1}, respectively) were added into the HAc/NaAc buffer (0.5 M, pH 4.5), and DMPO was selected as spin trapping agent (50 mM).

ABTS free radical scavenging assays**:** The free radical scavenging capacity was conducted using the ABTS radical cation scavenging capacity assay kit (Beijing Solarbio Science & Technology Co., Ltd), based on the reduction of •$ABTS^+$ radicals by TPNS. The •$ABTS^+$ working solution was prepared according to the manufacturer's instructions. Then, different concentrations of TPNS (2–10 μg mL^{-1}) were added to the diluted •$ABTS^+$ working solution. After 1 h of incubation at room temperature, the •$ABTS^+$ scavenging efficiency of TPNS was determined by measuring the absorbance at 734 nm.

DFT Calculations**:** DFT calculations have been employed to explain the redox-mediated ROS scavenging mechanism of TPNS. We have employed the first-principles to perform all Spin-polarization density functional theory (DFT) calculations within the generalized gradient approximation (GGA) using the Perdew-Burke-Ernzerhof (PBE) formulation. We have chosen the projected augmented wave (PAW) potentials to describe the ionic cores and take valence electrons into account using a plane wave basis set with a kinetic energy cut-off of 450 eV. Partial occupancies of the Kohn−Sham orbitals were allowed using the Gaussian smearing method and a width of 0.05 eV. The electronic energy was considered self-consistent when the energy change was smaller than 10^{-6} eV. A geometry optimization was considered convergent when the energy change was smaller than 0.05 eV $Å^{-1}$. Finally, the adsorption energies (E_{ads}) were calculated as $E_{ads}= E_{ad/sub} - E_{ad} - E_{sub}$, where $E_{ad/sub}$, E_{ad}, and E_{sub} are the total energies of the optimized adsorbate/substrate system, the adsorbate in the gas phase, and the clean substrate, respectively. The free energy was calculated using the equation: $G = E + ZPE – TS$, where G, E, ZPE and TS are the free energy, total energy from DFT calculations, zero point energy and entropic contributions (T was set to be 300K), respectively.

Cell culture**:** The immortalized proximal tubule epithelial (HK-2) cell line was purchased from the

American Type Culture Collection (Manassas, VA, USA). The HK-2 cells were cultured in Keratinocyte-SFM (Gibco, Rockville, MD, USA) supplemented with recombinant epidermal growth factor (0.5 ng mL^{-1}) and bovine pituitary extract (25 μg mL^{-1}) at 37 °C in an incubator supplied with a humidified atmosphere with 5% CO_2.

***In vitro ROS scavenging using TPNS*:** HK-2 cells were seeded into a 96-well plate at 5000 cells per well and incubated at 37 °C for 24 h under 5% CO_2. Then, TPNS with different concentrations (0, 5, 10 μg mL^{-1}) were added to culture media, and after 30 min incubation, cells were treated with 250 μM H_2O_2. After 24 h incubation under 5% CO_2 at 37 °C, cell viability was determined by cell counting kit-8 (CCK-8, Dojindo, Japan) assay.

2, 7-Dichlorofluorescin diacetate (DCFH-DA, Beijing Solarbio Science & Technology Co., Ltd), an oxidation sensitive fluorescent dye, was used to detect the intracellular ROS level. The non-fluorescent DCFH could be oxidized by the intracellular ROS to fluorescent DCF. Therefore, the quantity of intracellular ROS is correlated with the fluorescent intensity of DCF. After the aforementioned incubation with H_2O_2 for 24 h, cells were gently rinsed thrice with serum-free medium to remove the free TPNS. Then, a final concentration of 10 μM of DCFH-DA in serum-free medium was added to the cells and incubated in dark at 37 °C for 30 min. Afterwards, the cells were washed with serum-free medium thrice to remove unloaded DCFH-DA probe, then were imaged using a fluorescence microscope (Zeiss OBSERVER D1/AX10 cam HRC, Germany). To quantify the intracellular ROS levels, the cells were collected and rinsed, and the fluorescence of resuspended cells was evaluated by flow cytometer (Beckman Coulter, Brea, CA). Oxidative stress biomarkers-Malondialdehyde (MDA), hydrogen peroxide (H_2O_2), superoxide dismutase (SOD) and glutathione reductase (GSH) were measured by related kits purchased from Nanjing Jiancheng Bioengineering Institute.

Furthermore, cells seeded in 6-well plate were stained with Annexin V-FITC/PI apoptosis detection kit (Dojindo Molecular Technologies, Gaithersburg, MD) to detect the ratio of apoptotic cells. In brief, after the aforementioned incubation with H_2O_2 for 24 h, the cells were collected, washed twice with cold PBS. Next, cells were re-suspended with 4 °C PBS and mixed with 5 μL

Annexin V-FITC and 5 μL PI. Finally, after incubation in dark for 15 min, cells were analyzed by flow cytometry (Beckman Coulter, Brea, CA).

Measurement of mitochondrial membrane potential**:** After the aforementioned incubation with H_2O_2 for 24 h, cells were gently rinsed thrice with serum-free medium to remove the free TPNS, and then incubated with culture medium containing 1.0 μg/mL of JC-1 dye for 20min. The cells were washed with serum-free medium, incubated in fresh medium and visualized under a fluorescence microscope (Zeiss OBSERVER D1/AX10 cam HRC, Germany). JC-1 dyes were excited at 488 nm for JC-1 monomers and 585 nm emission for JC-1 aggregates.

In vitro biocompatibility evaluation of TPNS**:** The hemolysis assay was carried out to evaluate the red blood cells (RBCs) compatibility of TPNS. The experiments were approved and performed by the research ethical committee (No. 2019-784) of West China Hospital, Sichuan University, and all the experiments were performed in compliance with the relevant laws and national guidelines (GB/T 16886.4-2003/ISO 10993-4:2002, General Administration of Quality Supervision, Inspection and Quarantine of the People's Republic of China, Standardization Administration of the People's Republic of China). Informed consent was obtained for any experimentation with human subjects, and all regulations (e.g. IRB) were fulfilled for using human blood. In brief, 2 mL of whole blood was centrifuged for 10 min at 1000 rpm to collect RBCs and gently washed thrice with PBS solution. Then, 10 μL of diluted RBCs suspension was mixed with 990 μL TPNS dispersion at various concentrations (1-100 μg mL^{-1}). The mixed dispersions were incubated for 3 h at 37 °C and then centrifuged at 3500 rpm for 5 min before observing and recording the hemolysis phenomenon. The hemolysis ratio was quantified by measuring the absorbance value of supernatant at 540 nm with a microplate reader. Deionized water and PBS solution were used as the positive and negative control, respectively. Furthermore, after incubation for 3 h, 50 μL of the mixed dispersions were added to paraformaldehyde fixative. The fixed dispersions were centrifuged for 5 min at 3500 rpm, and then re-suspended with 1 mL deionized water. 50 mL of suspended RBCs dispersion was casted on RBCs smear, and was images using a 3D laser scanning microscope (VK-150K, EYENCE, Japan).

The cytotoxicity of TPNS was determined by the CCK-8 assay in vitro. Briefly, HK-2 cells were seeded into 96-well culture plates at the density of 5000 cells per well and incubated at 37 °C in an incubator with 5% CO_2 for 24 h. Afterwards, the cell culture medium was aspirated and fresh culture media containing various concentrations of TPNS (0–50 μg mL^{-1}) were added. After 24 incubation, cells were gently washed twice with sterile PBS and then treated with 100 μL fresh culture medium and 10 μL CCK-8 solution, and further incubated at 37 °C for 2 h. The cell viability was then quantified by measuring the absorbance value at 450 nm by a microplate reader (Bio-Rad, Hercules, CA).

***Biodegradability evaluation of TPNS*:** Mixtures contained 50 μg of myeloperoxidase from human neutrophils (hMPO, Athens Research and Technology) with an activity of 1.1 U μg^{-1} per 500 μg of TPNS suspended in 500 μL of 20 mM PBS. Hydrogen peroxide (H_2O_2) was added at a rate of 200 mM per hour for 5 h. As a result of the loss of activity of hMPO in the PBS, the hMPO was replenished every 5 h and the incubation system was maintained at 37 °C for 24 h.

To investigate the intracellular degradation of TPNS, HK-2 cells coincubated with TPNS (50 μg mL^{-1} in DMEM medium) for 0.5, 1, and 2 d were washed with PBS and then collected and melted by lysis buffer. The residual Ti contents in HK-2 cells were determined by ICP-MS.

To investigate the in vivo degradation and metabolism process, the urine and feces of mice were collected at different time points (6, 12, and 24 h) after intravenous injection of TPNS into normal mice. The Ti contents in urine and feces were quantitatively determined by ICP-MS.

***Acute kidney injury (AKI) models in mice*:** Eight-week-old C57BL/6 mice were purchased from Chengdu Dashuo Experimental Animal Co. LTD (Chengdu, China). Mice were housed at the Animal Experiment Center of West China Hospital, Sichuan University (Chengdu, China). All mice were maintained under specific pathogen-free conditions. All animal treatments were conducted according to the "Guide for the Care and Use of Laboratory Animals" (8th Edition. 2011), and AVMA Guidelines for the Euthanasia of Animals (2013 Edition) and were approved (No. 2018169A) by the Institutional Animal Care and Use Committee of Sichuan University West China hospital. For AKI

model establishment, mice were deprived of water but given free access to food for 15 h. After water deprivation, the two hindlimbs of mice were equally intramuscularly injected with 50% glycerol at a dose of 8 mL kg^{-1}. After that, all the mice had free access to water and food. Symptoms of AKI, such as a lack of activities and decreased urine output, could be observed in a few hours after glycerol injection.

Biodistribution of TPNS in AKI mice**:** Fluorescent labeled TPNS were prepared by adding 10 mg/mL of Cy5.5 to the TPNS solution at 25 °C for 5 h. The excess dye molecules were removed by centrifugation and washed away with PBS thrice until no noticeable color change was observed from the supernatant fluids followed by resuspension in PBS. AKI mice were intravenously injected with the Cy5.5-labelled TPNS at a dose of 500 μg/kg. At different time points (5min, 1, 3, 7, 12), mice were sacrificed to harvest major organs including heart, liver, spleen, lung, and kidney, and the fluorescence images of these organs were obtained by a fluorescence (Caliper Life Sciences, IVIS Spectrum) imaging system.

Treatment of AKI mice**:** Two hours after the AKI model induction, different treatments were performed on AKI model mice: group 1 was healthy mice treated with PBS (n = 5); group 2 was healthy mice (n = 5) treated with TPNS (5 μg in 100 μL PBS), group 3 was AKI mice treated with PBS (PBS, n = 5); group 4 was AKI mice treated with TPNS (5 μg in 100 μL PBS, n = 5). The TPNS was administrated once. The survival curve were monitored for 2 weeks after treatment. The body weight variations in each group after treatment were monitored for 24 h.

Kidney function test**:** Kidney function tests were performed to evaluate the treatment of AKI. After 24 h post injection, mice were sacrificed to collect blood samples for detecting the BUN and CRE levels using the corresponding detection kits according to the manufacturer's instructions.

Renal histology and immunofluorescence**:** Kidney tissues were collected 24 h after the model induction and fixed with paraformaldehyde (4% in PBS), embedded in paraffin wax and cut into 5 μm slices for following hematoxylin-eosin (H&E) staining, TUNEL fluorescent staining, and

immunofluorescence staining. H&E, TUNEL and immunofluorescence staining were performed according to standard manufacturer's instruction protocols. Sections were deparaffinized with xylene and rehydrated in graded ethanol. Sections were microwaved in 10 mM sodium citrate and then incubated with primary antibodies overnight at 4 °C and the secondary antibodies for 30 min at 37 °C.

***Analysis of renal tissues after treatment*:** Kidneys from each group were frozen and stored at -80 °C until the assay. Kidney homogenates were prepared according to the protocols of different assays. H_2O_2, MDA, SOD, GSH, and LDH level were assessed with corresponding assay kits purchased from Nanjing Jiancheng Bioengineering Institute. KIM-1 expression and HO-1 expression were measured with KIM-1 or HO-1 ELISA kits (Thermo Scientific, USA). DNA damage was evaluated with 8-OHdG ELISA kit (Thermo Scientific, USA). The inflammatory factors expression were determined with related ELISA kits (Thermo Scientific, USA).

***Confocal imaging of ROS production in kidneys*:** To assess superoxide production histologically, kidneys of mice were collected and stored in optimum cutting temperature (O.C.T.) specimen matrix for cryostat sectioning at -20 °C. The frozen kidneys were further sectioned into 5 μm tissue slices. Frozen kidney tissue slice were washed with PBS and stained with 1 mM dihydroethidium (DHE) for 30 min to detect ROS formation. Then a cover glass was applied to each slide and confocal imaging was performed using a Nikon A1R confocal microscope.

***In vivo toxicity assessment*:** Healthy mice were randomized into two groups: group 1 was healthy mice intravenously injected with 100 µL PBS as the control group (n = 5), the other group was healthy mice intravenously injected 5 µg of TPNS in 100 µL PBS (n = 5). The mice were euthanized at 24 h post-injection. Subsequently, the major organs (heart, liver, spleen, lung, and kidney) and blood samples were harvested. Histological changes in organs were analyzed by H&E staining, and whole blood samples were used for hematology analysis. The plasma of blood samples was collected after centrifugation at 2000×*g* for 15 min at 4 °C, and the serum biochemistry test included two important indicators of hepatic function as aspartate aminotransferase (AST) and alanine aminotransferase

(ALT), and two indicators of kidney function as blood urea nitrogen (BUN) and creatinine (CRE). Serum TNF-α and IL-6 levels were quantified by the ELISA assay.

***RT-PCR array*:** For PCR analysis, total RNA was isolated with an RNA extraction Kit (Vazyme, China) according to the manufacturer's protocol. RNA (1 μg) was transcribed into cDNA using reverse transcriptase (Vazyme, China). Each PCR array was a 96-well plate containing gene specific optimized real-time PCR primer sets for 90 genes related to NF-κB signaling pathway. All treatments were performed in triplicate to calculate statistical significance and the results were calculated using the 2-ΔΔCt method. Genes with fold-changes more than 2 or less than 1/2 were considered to have biological significance.

***Western blot analysis*:** The total proteins from kidney tissue were extracted using ice-cold RIPA lysis buffer containing phosphatase and protease inhibitor cocktail (Beyotime, China). The concentrations of extracted proteins were determined using a BCA protein assay kit (Biosharp, China). An equal amount of protein from each sample was run in 10% SDS-PAGE gel, then transferred to polyvinylidenedifluoride (PVDF) membranes (Millipore, USA). PVDF membranes were blocked with 5% skim milk at room temperature for 2 h and then incubated with primary antibodies at 4 °C overnight, followed by incubation with secondary antibodies (1:1000, Abcam, UK) for 1 h at room temperature. The intensity of bands was visualized and determined using a ChemiDoc™ XRS detection system (Bio-Rad, USA). Primary antibodies used were: Drp-1 (1/3000, Novus, USA), Opa-1 (1/3000, Novus, USA), NF-κB p65 (1/1000, proteintech), p-NF-κB p65 (Ser536) (1/500, CST, USA), Bax (1/5000, proteintech, USA), Bcl-2 (1/1000, proteintech, USA), Cyto-C (1/2000, proteintech, USA), Cleaved Caspase-3 (1/1000, CST, USA), TNF-α (1/2000, Abcam, UK), IL-6 (1/1000, CST), and GAPDH (1:1000, Abcam, UK).

***Statistical Analysis*:** Quantitative data were presented as mean ± standard deviation (mean ± SD). One-way analysis of variance was performed to determine the statistical differences; differences were accepted as significant at a P-value lower than 0.05.

Supporting Results

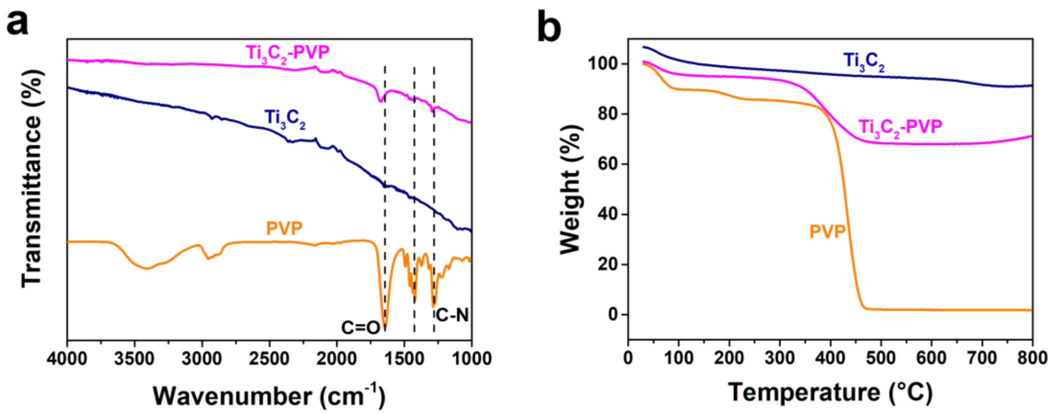

Figure S1. a) FTIR spectra of Ti_3C_2, PVP, and Ti_3C_2-PVP, indicating the successful surface modification of Ti_3C_2 with PVP. b) TGA curves of Ti_3C_2, PVP, and Ti_3C_2-PVP.

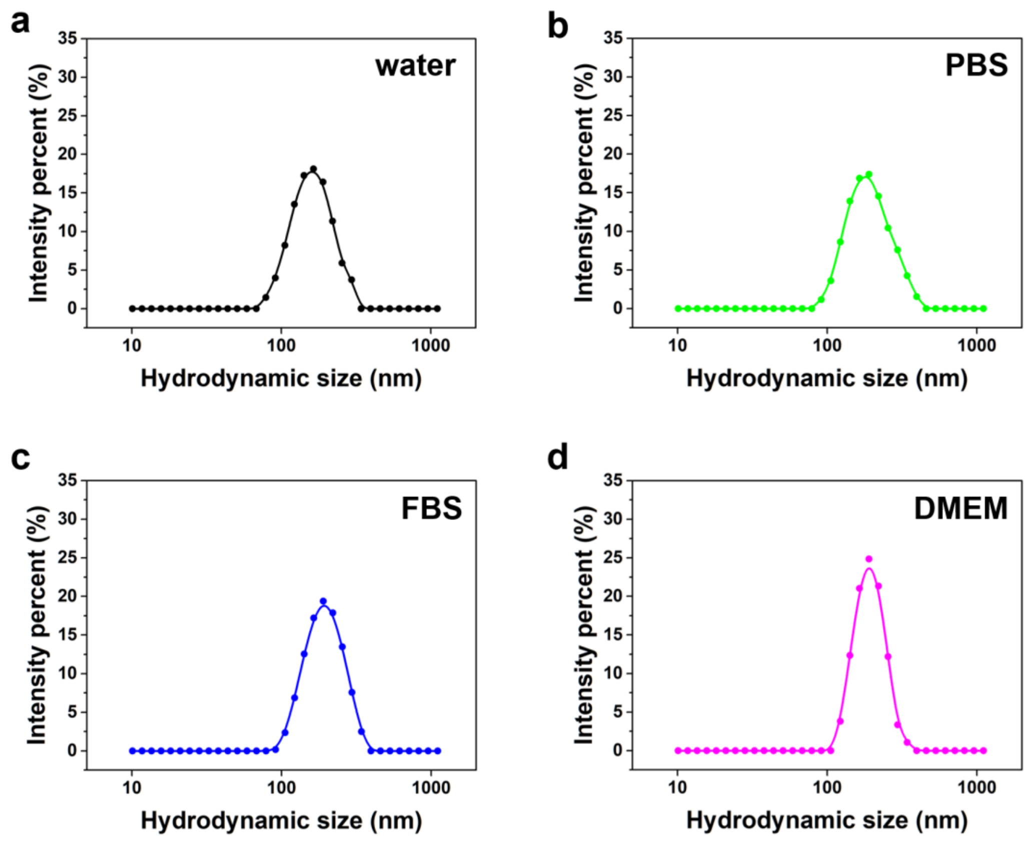

Figure S2. Dynamic light scattering analysis of TPNS in water, PBS, FBS, and DMEM after 7 days.

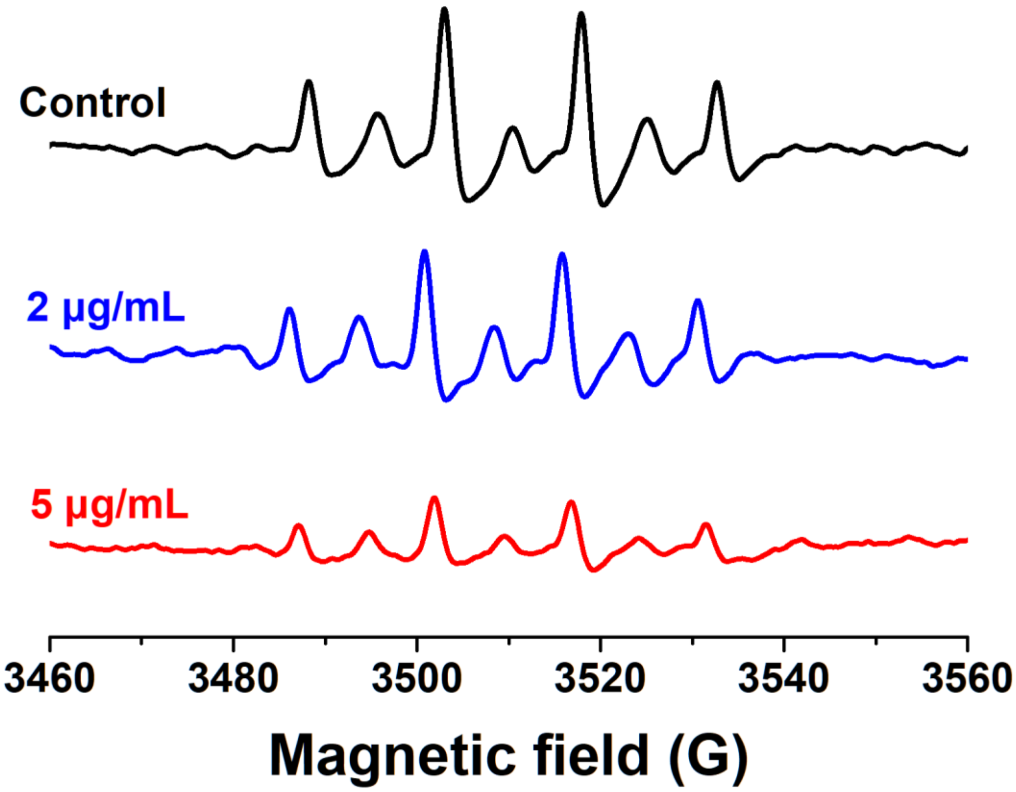

Figure S3. •OH scavenging of TPNS determined by ESR.

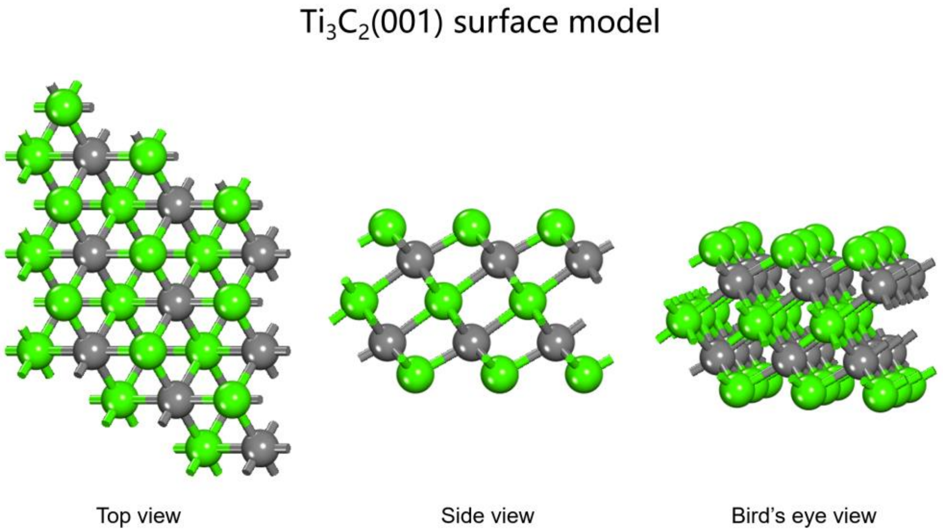

Figure S4. Geometrically optimized single-layer Ti_3C_2 nanostructure observed from different angles.

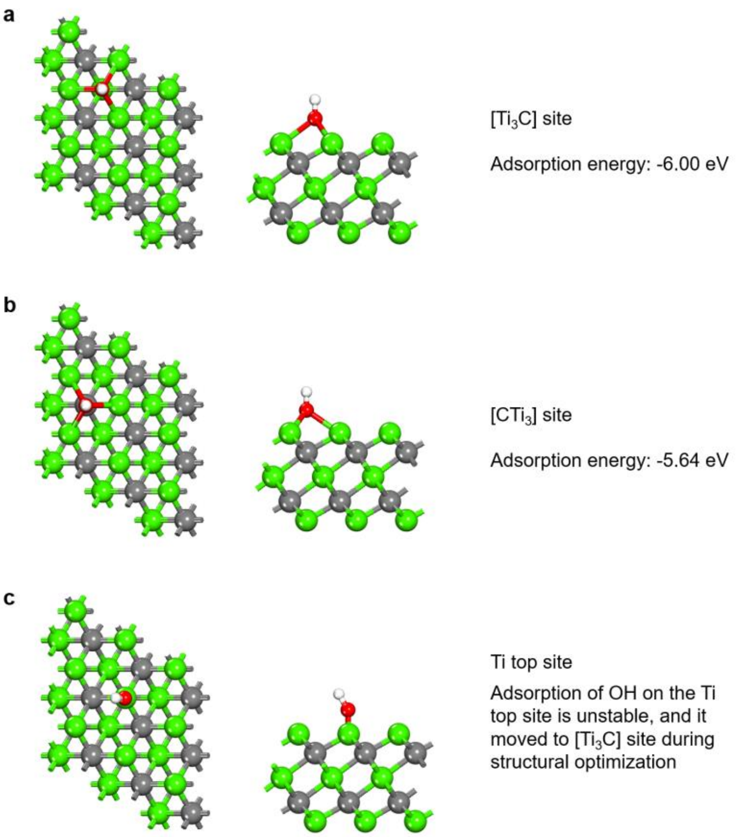

Figure S5. Three different •OH adsorption sites onto Ti3C2 with optimized adsorption energy.

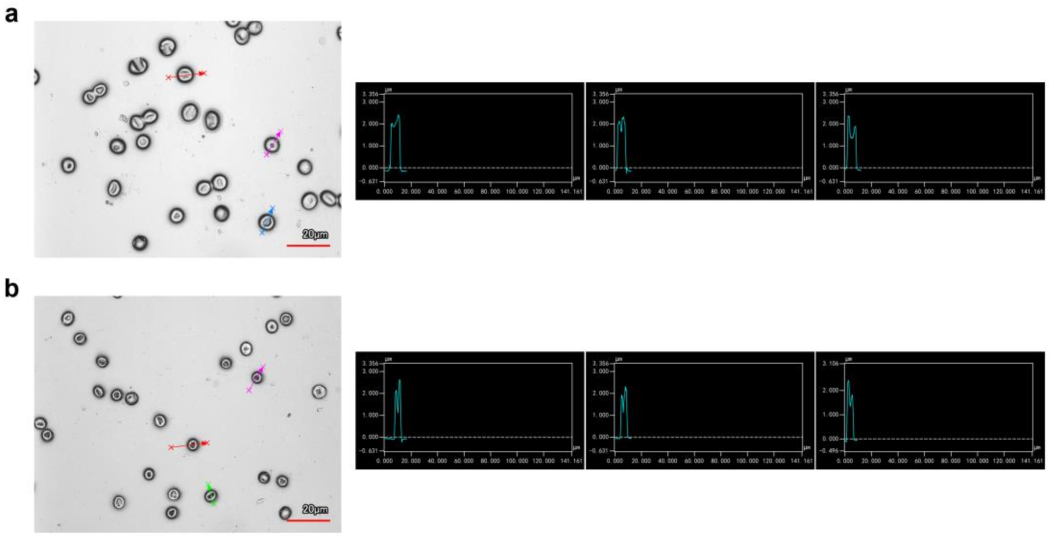

Figure S6. The 3D microscope images and corresponding outline size of single cell for RBCs before (a) and after (b) incubation with TPNS.

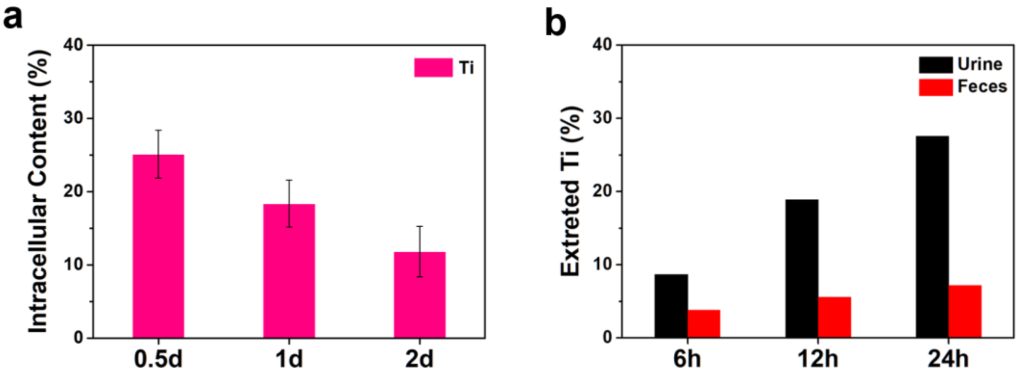

Figure S7. (a) Intracellular Ti content in HK-2 cells after the co-incubation of TPNS with HK-2 cells for different incubation durations (0.5, 1, and 2 d). (b) Accumulated Ti (in urine and feces) excretion out of the mice body after the administration of TPNS for different durations (6 h, 12 h and 24 h).

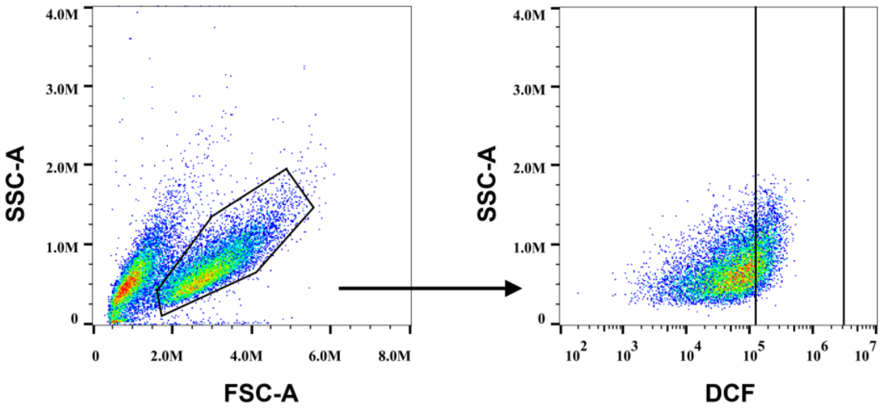

Figure S8. Gating strategy to determine the intracellular ROS level presented in Figure 4c.

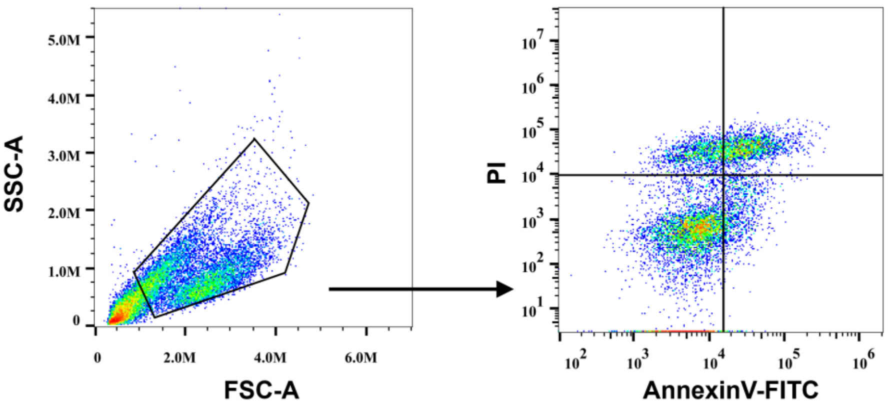

Figure S9. Gating strategy to determine the cell death ratios presented in Figure 4f.

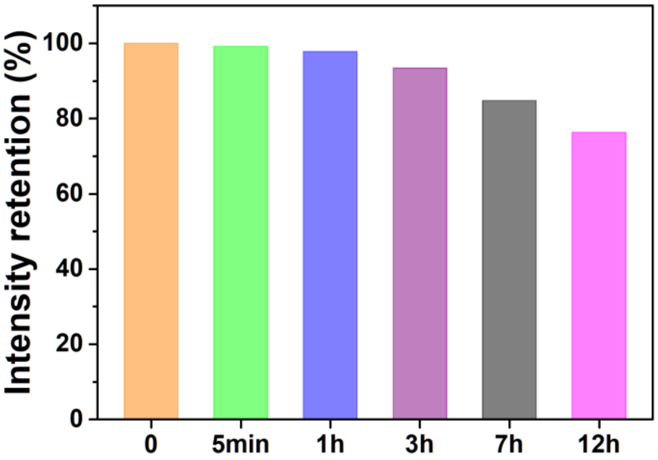

Figure S10. The fluorescence intensity retention of cy5.5-TPNS in solution at different time.

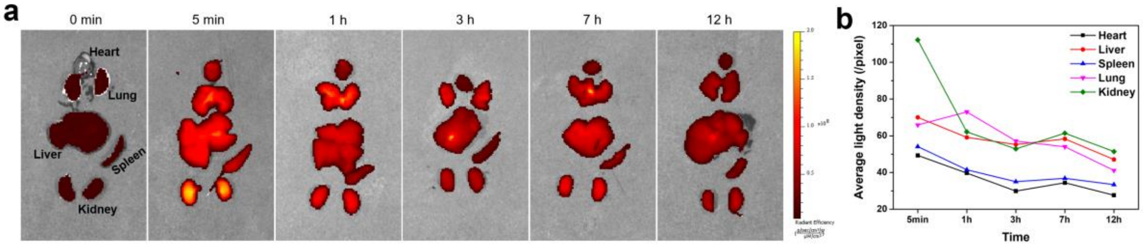

Figure S11. a) Fluorescence signal of cy5.5-TPNS in heart, liver, spleen, lung, and kidney. b) The semi-quantitative results of fluorescence images of main organs.

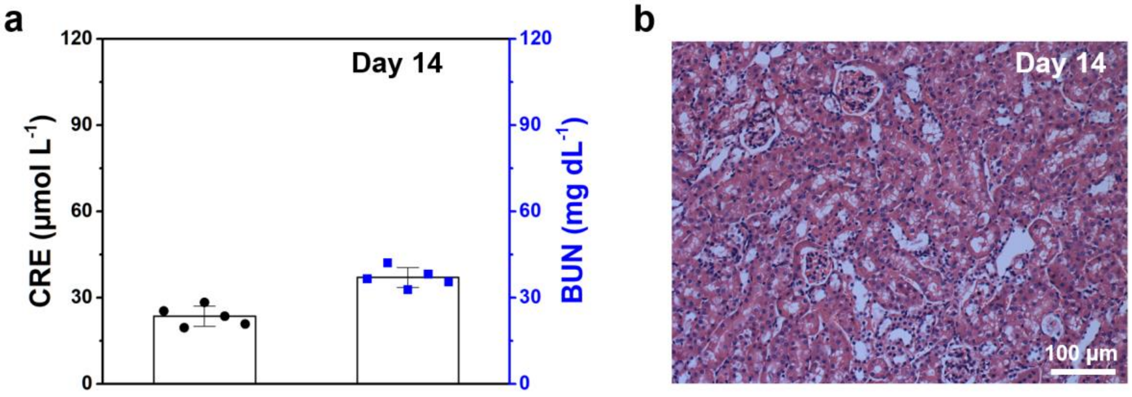

Figure S12. a) CRE and BUN levels in serum from AKI mice treated with TPNS after 14 days of

treatment. b) H&E-stained renal tissue from AKI mice treated with TPNS after 14 days of treatment.

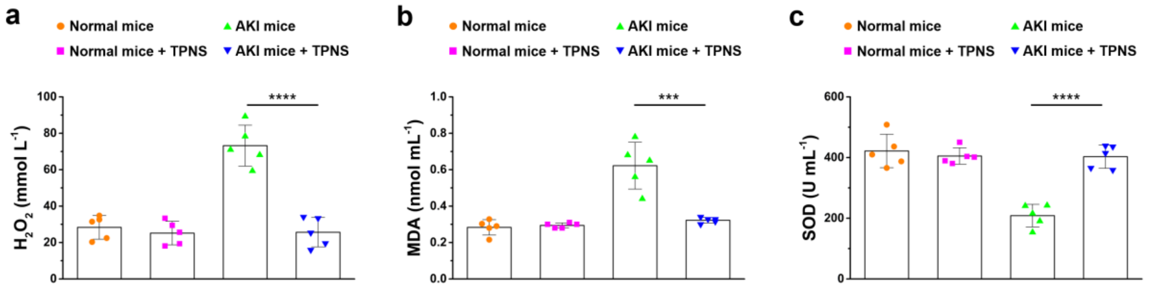

Figure S13. a) H_2O_2, b) MDA, and c) SOD levels in the blood serum from AKI mice at 24 h after different treatment. Statistical significance compared with the AKI group is shown (***P < 0.001; ****P < 0.0001). All values are expressed as means ± SD, n=5.

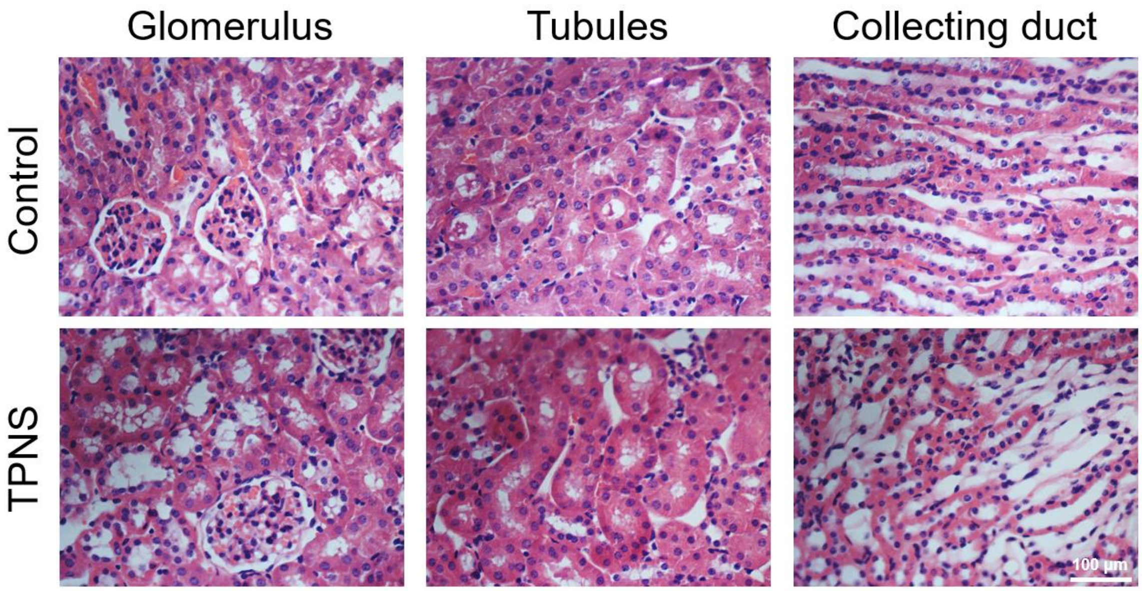

Figure S14. H&E staining of renal tissues for healthy mice and TPNS-treated mice.

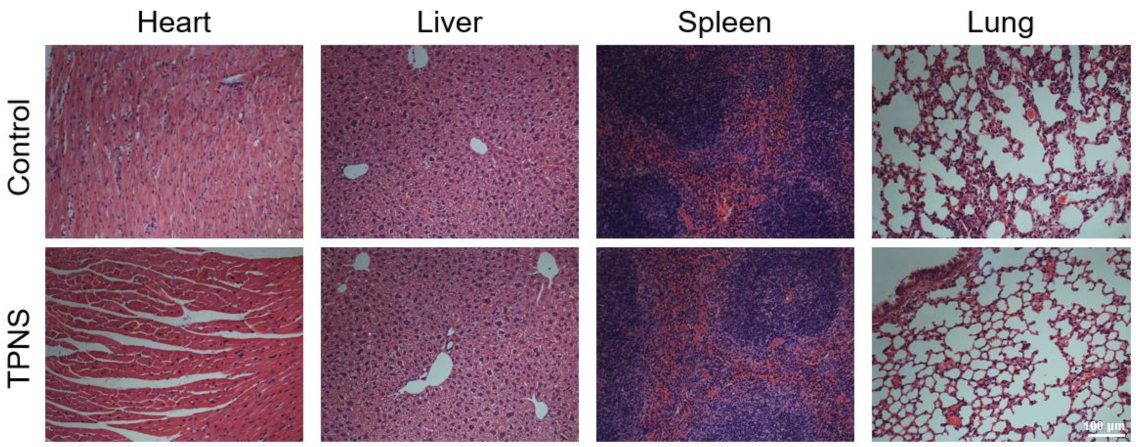

Figure S15. Evaluation of in vivo toxicity of TPNS to major organs (heart, liver, spleen, and lung) at 24 h after intravenous administration.

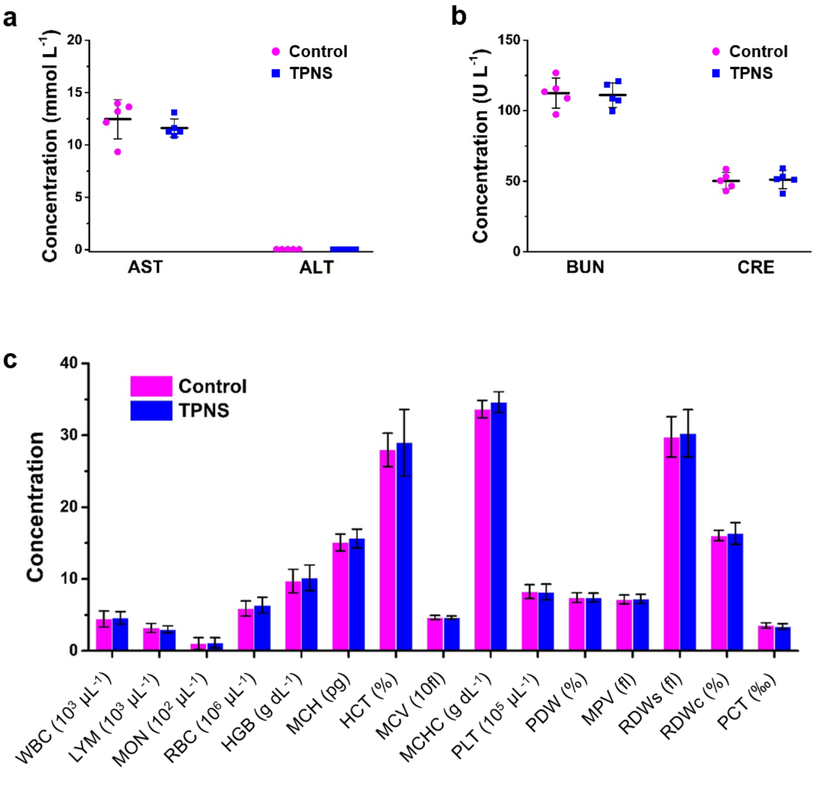

Figure S16. a) Serum levels of liver function indicators: aspartate transaminase (AST) and alanine transaminase (ALT). b) Serum levels of kidney function indicators: blood urea nitrogen (BUN) and creatinine (CRE). c) Hematology analysis of whole-blood parameters in healthy mice treated with PBS or TPNS. All values are expressed as means ± SD, n=5.

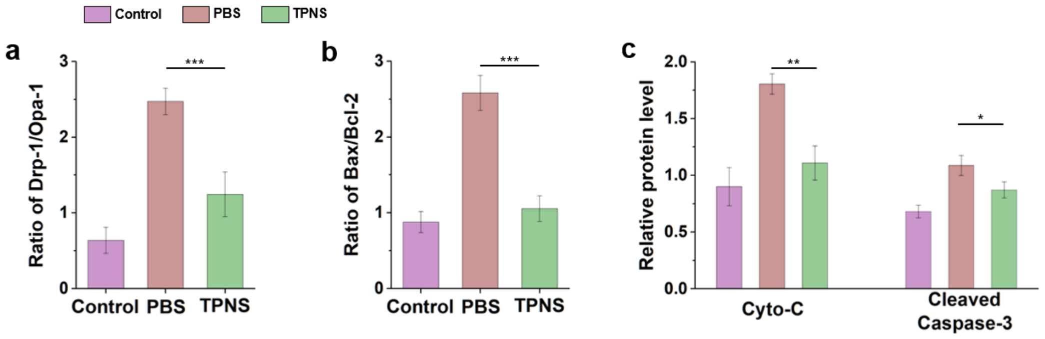

Figure S17. Quantitative expression levels of a) Drp-1/Opa-1, b) bax/Bcl-2, c) Cyto-C and Cleaved Caspase-3 in renal tissues from different groups. Statistical significance compared with the PBS-treated AKI group is shown (*P < 0.05; **P < 0.01; ***P < 0.001). All values are expressed as means ± SD, n=3.

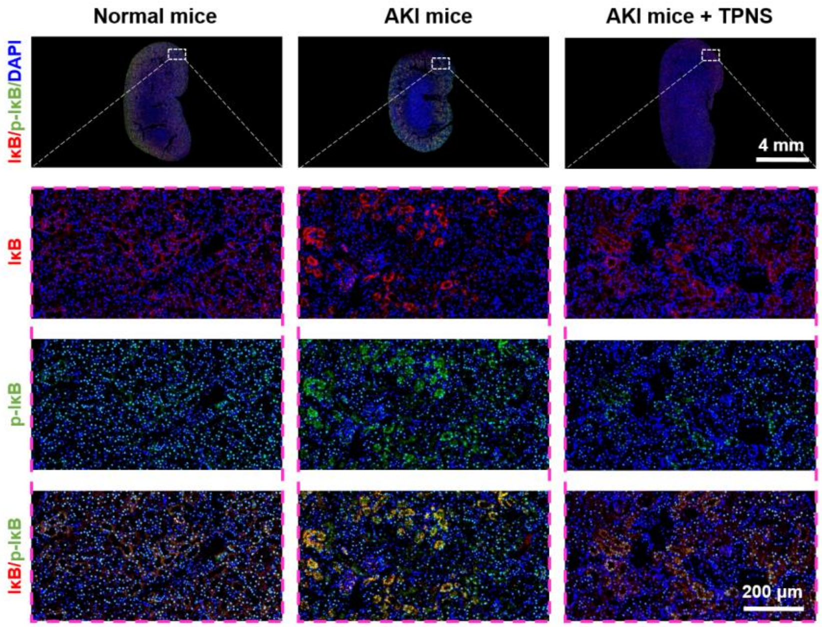

Figure S18. Immunofluorescence staining of IκB level in kidney sections from different groups.

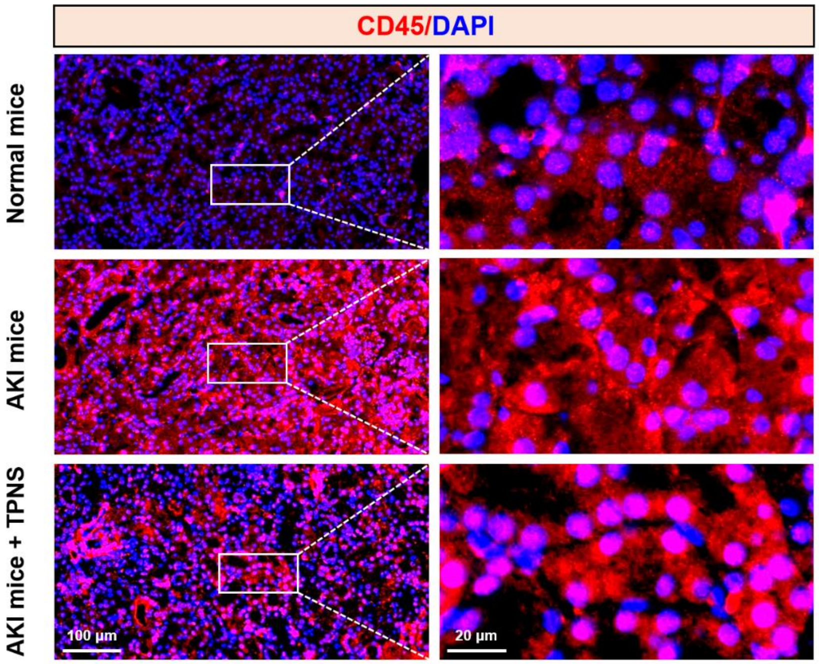

Figure S19. Immunofluorescence staining of CD45 level in kidney sections from different groups.

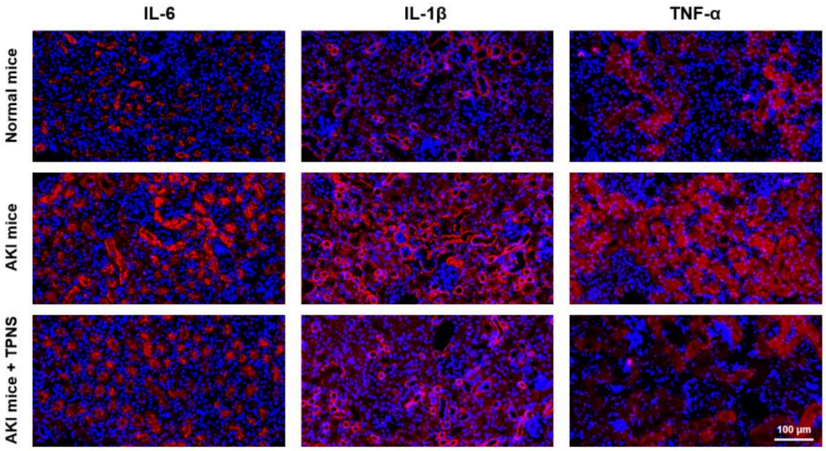

Figure S20. Immunofluorescence staining of inflammatory factors in kidney sections from different groups.
